# Analysis of Variance in Neuroreceptor Ligand Imaging Studies

**DOI:** 10.1371/journal.pone.0023298

**Published:** 2011-08-17

**Authors:** Ji Hyun Ko, Anthonin Reilhac, Nicola Ray, Pablo Rusjan, Peter Bloomfield, Giovanna Pellecchia, Sylvain Houle, Antonio P. Strafella

**Affiliations:** 1 PET Centre, Centre for Addiction and Mental Health, University of Toronto, Toronto, Ontario, Canada; 2 Toronto Western Hospital and Research Institute, University of Toronto, Toronto, Ontario, Canada; 3 Department of Life Sciences, Australian Nuclear Science and Technology Organization, Lucas Heights, New South Wales, Australia; University of Texas, M.D. Anderson Cancer Center, United States of America

## Abstract

Radioligand positron emission tomography (PET) with dual scan paradigms can provide valuable insight into changes in synaptic neurotransmitter concentration due to experimental manipulation. The residual t-test has been utilized to improve the sensitivity of the t-test in PET studies. However, no further development of statistical tests using residuals has been proposed so far to be applied in cases when there are more than two conditions. Here, we propose the residual f-test, a one-way analysis of variance (ANOVA), and examine its feasibility using simulated [^11^C]raclopride PET data. We also re-visit data from our previously published [^11^C]raclopride PET study, in which 10 individuals underwent three PET scans under different conditions. We found that the residual f-test is superior in terms of sensitivity than the conventional f-test while still controlling for type 1 error. The test will therefore allow us to reliably test hypotheses in the smaller sample sizes often used in explorative PET studies.

## Introduction

Positron emission tomography (PET) is a widely used research tool to assess the neurochemical changes induced by pharmacological, behavioral or physiological intervention. Researchers measure changes in binding potentials (BP) of radioactive tracers to receptor sites, which is thought to reflect the synaptic concentration of the targeted neurotransmitter [1]. Aston et al. [2] established a robust voxel-wise method to test hypotheses with PET receptor parametric mapping, i.e., the residual t-test. By utilizing the residuals of the fitting in the simplified reference tissue model [3], this method greatly increases the degree of freedom that determines the size of the t-statistic considered significant. This is important because low sample sizes, and therefore small degrees of freedom (and as a result, low statistical power) are one of the major obstacles in costly PET imaging studies. Aston et al.'s method has been widely used in many radio-ligand PET studies with repeated measures designs [4]–[12].

However, when there are more than two conditions, if we are to take advantage of the large degree of freedom of the residual method proposed by Aston et al. [2], we are limited to performing multiple t-tests, thus increasing our chances of making a Type 1 error (i.e., false positive). In such cases, an analysis of variance (ANOVA) performed prior to the t-tests would limit this problem. In order to retain the benefits of using the residuals however, the ANOVA (f-test) must first be converted into the “residual f-test”.

Here, we propose the use of the residual f-test for testing hypotheses when there are more than two conditions. In order to demonstrate its usefulness, we simulated a series of [^11^C]raclopride PET images with different BPs. We then compared the number of voxels showing a significant f-statistic when tested with the conventional f-test, using SPM2 (Wellcome Department of Cognitive Neurology, London, UK; http://www.fil.ion.ucl.ac.uk/spm/), and when tested with the residual f-test. In addition, our previously published data [9] was re-visited with the proposed residual f-test to confirm its usefulness in reality.

## Materials and Methods

### Theory

Under the same assumption stated in Aston et al. [2], i.e., 1) the residuals are not correlated in time; 2) the basis function fitting method [3] is equivalent to a nonlinear least squares fit; 3) The only sources of differences in parameter estimates among the scans are the noise of the PET measurement and the biological effect of different condition used in the PET scans; 4) The noise in the reference tissue is negligible, if subjects underwent multiple numbers of scans in different conditions, we can test the effect of condition using:
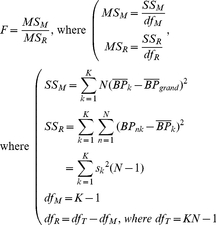
(1)where *K* is the number of conditions and *N* is the number of subjects. *MS_M_* and *MS_R_* are mean squares of the model and the residuals, respectively. *SS_M_* and *SS_R_* are sums of squares of the model and the residuals, respectively. *df_M_* and *df_R_* are the degrees of freedom of the model and the residuals (NB the “residuals” we are referring to here relate only to the calculation of *MS_R_*, and are different from the residuals from the kinetic model used for the “residual f-test”), respectively. *BP_nk_* is estimated by a single PET image from a subject (*n*) in condition (*k*). 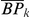 is the mean BP of all subjects over condition (k) and 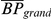 is the mean BP over all images. As explained by Aston et al. [2], the estimated dispersion matrix for all parameters [13] is :
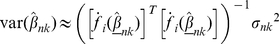
(2)where 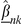 is a vector of the parameter estimates (i.e., 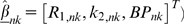), derived from the compartmental model fit of the PET data [3], where 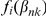 is the value of the operational equation (Eq. 7 of Lammertsma and Hume [14]) at the *i*-th time point.
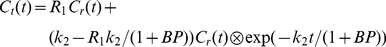
(3)
*C_t_* and *C_r_* is the concentration of radioligand in the tissue and the reference region, respectively. *R_1_* is the ratio of plasma to tissue transport constant between tissue and reference region, *k_2_* is the plasma efflux constant.

Using the derivatives of the operational equation (Eq. 3), the variances, *σ_nk_^2^*, can be estimated by
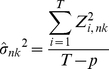
(4)Where *T* is the number of frames of PET images and *p* is number of parameters (*R_1_*, *k_2_*, *BP*). *Z_i_* is the residuals of the least square fit of the operational equation (Eq. 3) at the *i*-th time point.

Therefore, the residual F-statistics (*F_residual_*) is:
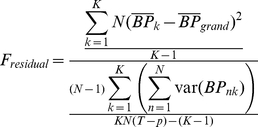
(5)Where var(*BP_nk_*) is taken from the estimated dispersion matrix of *BP_nk_* [Eq 2 and 4]. As a result, the degree of freedom in equation (Eq. 5) is increased compared to the standard f-test [K−1, KN−K] by an order of magnitude proportional to the number of PET frames [K−1, KN(T−p)−(k−1)].

### Simulation

We simulated realistic four dimensional [^11^C]raclopride PET images in two steps [2]. First, realistic time-activity-curves (TACs) reflecting intervention-induced displacement of dopamine were simulated using Matlab (R14SP3) from the real reference region TAC obtained from [^11^C]raclopride PET images for each of the ten individuals acquired during our previous study [9]. The tissue TACs were simulated using Eq. 3.

In order to reflect the intervention-induced focal changes in [^11^C]raclopride binding to dopamine receptors in the left caudate nucleus, BP was varied for 0%, −5%, −10% and −20% from 3.0 while *R_1_* was set to 1.39. TACs were also simulated for the rest of the striatum (BP = 3.0, *R_1_* = 1.39), grey matter (BP = 0.1, *R_1_* = 1.0), white matter (BP = 0, *R_1_* = 1.0), CSF (BP = 0, *R_1_* = 1.2), skin/skull/muscle (BP = 0, *R_1_* = 0.3). *k_2_* is set to 0.37 min^−1^. For the cerebellum, real cerebellar TACs were used [9]. The parameters for the striatum are employed from Farde et al. [15]. For other regions, the parameters are employed from our previous real PET data [9].

**Figure 1:**
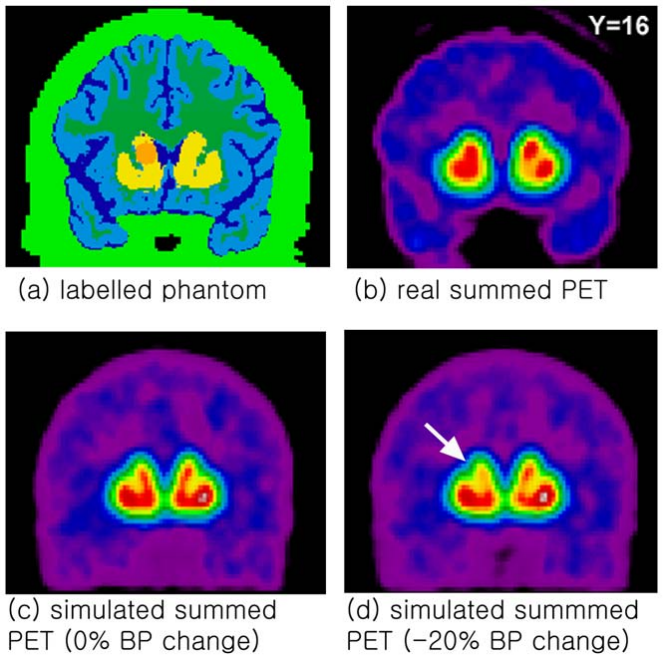
Real and simulated PET images. (a) A labeled MRI of a single subject. High-resolution MRI (GE Signa 1.5 T, T1-weighted images, 1 mm slice thickness) of each subject's brain was acquired and transformed into standardized stereotaxic space [16] using automated feature-matching to the MNI template [17], then segmented for striatum (yellow), grey matter (light blue), white matter (dark green), CSF (dark blue), skin/skull/muscle (light green), and cerebellum (not shown) [18]. A spherical region-of-interest in the left caudate nucleus was manually defined at X = −12, Y = 16, Z = 8 (radius 6 mm, 99 voxels, orange). (b) Real summed PET image of a single subject. (c) Simulated summed PET image of a single subject with 0% changes in the left caudate BP. (d) Simulated summed PET image of a single subject with 20% decrease in the left caudate BP. The reduced signal is apparent in the left caudate nucleus (arrow).

High-resolution MRI (GE Signa 1.5 T, T1-weighted images, 1 mm slice thickness) of each subject's brain was acquired and transformed into standardized stereotaxic space [16] using automated feature-matching to the MNI template [17], then segmented for striatum, grey matter, white matter, CSF, skin/skull/muscle, and cerebellum [18]. The left caudate nucleus was manually defined as a sphere located at X = −12, Y = 16, Z = 8 (radius 6 mm, 99 voxels) (Fig. 1).

A total of 60 dynamic PET images (10 subjects×6 different BP-value for the left caudate, i.e., three 0%, one −5%, one −10%, one −20%) were simulated from the labeled MRIs from each subject [18] using PET–SORTEO [19], [20], configured for the Ecat Exact HR+ scanner operating in 3D mode. This simulator generates realistic data (emission and transmission projections) given a numerical phantom description, the scanner geometry and its physical characteristics. PET–SORTEO has been thoroughly validated for the geometry of the Ecat Exact HR+ scanner and its realistic noise representative of data collection process [19]. The simulated detection system is made of 32 crystal rings with 576 detection units each, allowing the simultaneous acquisition of 63 transverse planes of 56.2 cm each over an axial extent of 15.52 cm.

The simulation of the transmission acquisition for each numerical phantoms was performed following a standard 10 min 2-D acquisition protocol (span = 15, maximum ring difference (MRD) = 7, lower level discriminator (LLD) = 350, upper level discriminator (ULD) = 650) using the three rotating rod sources (^68^Ge, 200 Mbq each). Span and MRD refers to the geometry of the acquisition. LLD and ULD are the energy threshold values in between which the measured energy of a photon must lie in order to be accepted. The 3-D correction factors were then derived for each phantom from the simulated transmission scan in 3 steps. First, the 2-D attenuation correction factors were computed from the 2-D transmission data and a simulated blank scan. Then, an attenuation map was reconstructed from the 2-D factors and forward projected to generate the oblique correction factors, leading to the set of 3-D attenuation correction factors.

Each of the 60 dynamic emission scans was simulated from the numerical phantoms for the Ecat Exact HR+ scanner operating in 3-D mode (span = 9, mrd = 22, lld = 350, uld = 650), then normalized and corrected for randoms, scatter contamination, attenuation, dead-time, and radioelement decay and finally reconstructed using a standard 3D filtered back projection algorithm with the Hanning filter and a cutoff frequency of 0.5 mm. Each reconstruction yielded 29 time frames (6×1 min, 20×2 mins, 3×5 mins) of data volumes (128×128×63 voxels of 2.06×2.06×2.425 mm^3^ each).

Voxelwise [^11^C]raclopride BP was calculated using a simplified reference tissue (cerebellum) method [3], [14]. Durbin-Watson statistics [21] was evaluated to ensure the feasibility of using residuals for estimation of standard deviation [2].

In order to evaluate the sensitivity of the residual f-test, different combinations of three conditions were then chosen to undergo both the conventional f-test using SPM2 and residual f-test (Table 1). Residual f-statistics greater than 15.3 (n = 10) and 15.6 (n = 6) were considered significant, determined by resel correction (p<0.001 corrected) [22]. Conventional f-statistics determined by SPM2 were considered significant using family-wise-error (FWE; p<0.05 corrected).

**Table 1:** Sensitivity of different f-test from simulated [^11^C]raclopride PET.

		conventional f-test		residual f-test	
Change in BP	number of subjects	Max f	number of voxels (p<0.05, corrected)	Max f	number of voxels (p<0.001, corrected)
0, 0, 5%	6	15.2	0	18.1	2
0, 5, 10%	6	33.0	0	26.5	20
0, 0, 10%	6	52.2	1	33.5	44
0, 10, 20%	6	147.0	51	120.5	127
0, 0, 20%	6	199.3	46	147	151
0, 0, 5%	10	17.5	0	15.7	2
0, 5, 10%	10	32.7	2	22.9	12
0, 0, 10%	10	34.0	16	28.3	31
0, 10, 20%	10	162.6	101	107.1	112
0, 0, 20%	10	196.9	122	135.8	141

BP was modulated for 99 voxels in the left caudate nucleus (spherical VOI centered at X = −12, Y = 16, Z = 8 with 6 mm radius).

Max f reflects the highest f-value observed in the f-test parametric map.

Specificity, i.e., weather the f-statistics detects false positives, was also compared between the residual f-test and conventional f-test (Table 2).

**Table 2:** Specificity of different f-test from simulated [^11^C]raclopride PET.

		conventional f-test (corrected)		residual f-test (corrected)	
number of subject	p-threshold	f-threshold	number of voxels	f-threshold	number of voxels
6	p<0.05	*39.1	0	11.3	6
	p<0.01	55.9	0	13.0	2
	p<0.001	91.5	0	**15.6	0
					
10	p<0.05	*20.6	0	11.0	0
	p<0.01	26.4	0	12.6	0
	p<0.001	36.8	0	**15.3	0

Three conditions per subject have been included in the f-test. BP was not changed in all three conditions.

*f-statistics are considered significant at p<0.05 (corrected) for conventional f-test.

**f-statistics are considered significant at p<0.001 (corrected) for residual f-test.

### PET analysis

Previously published data [9] with [^11^C]raclopride PET was re-analyzed with the proposed one-way ANOVA (i.e., residual f-test). In brief, ten normal right-handed subjects (20–28 years; four males) underwent three scans on three different days after the procedures were approved by the local research ethics committee and informed consent was obtained. Continuous theta burst stimulation (cTBS) was applied to three different targets; the right dorsolateral prefrontal cortex (DLPFC), left DLPFC and vertex (control site) per each day. After cTBS, subjects performed the montreal card sorting task (MCST), which has previously been shown to induce striatal dopamine release compared to a control condition [11]. PET frames were summed, registered to the corresponding MRI [23] and transformed into standardized stereotaxic space using the transformation parameters previously determined for the MRI [17]. Voxelwise [^11^C]raclopride BP was calculated using a simplified reference tissue (cerebellum) method [3], [14]. Durbin-Watson statistics [21] was performed to ensure the feasibility of using residuals for estimation of standard deviation [2]. For statistical analysis, residual f-statistics greater than 15.3 were considered significant (p<0.001 corrected) [22]. Conventional f-statistics determined by SPM2 were considered significant using family-wise-error (FWE; p<0.05 corrected).

## Results

A summary of the simulation study is shown in Table 1. With 6 randomly selected subjects, the residual f-test successfully identified the significant effect of condition in the left caudate, while the conventional f-test did not, especially when there were less prominent changes in BP (i.e., 5%–10% change). When the changes in BP were more prominent (i.e., 20% change), the conventional f-test was also able to detect the significant effect, but less voxels were identified compared with the residual f-test, i.e., 46–51 voxels vs. 127–151 voxels, respectively. When 10 subjects were included in the analysis, both statistical tests identified a similar number of voxels as having significantly different BPs, with the residual f-test spotting slightly more voxels than the conventional f-test, i.e., 2–141 voxels vs. 0–122 voxels, respectively.

No false positives were detected in either method when no changes in BP were simulated (p<0.001 corrected) (Table 2). When a less stringent threshold was applied (i.e., p<0.05 or p<0.01), a few false positives were detected when using the residual f-test when only six subjects were included. The conventional f-test did not produce any false positives when corrected for multiple comparisons (Table 2).

**Figure 2:**
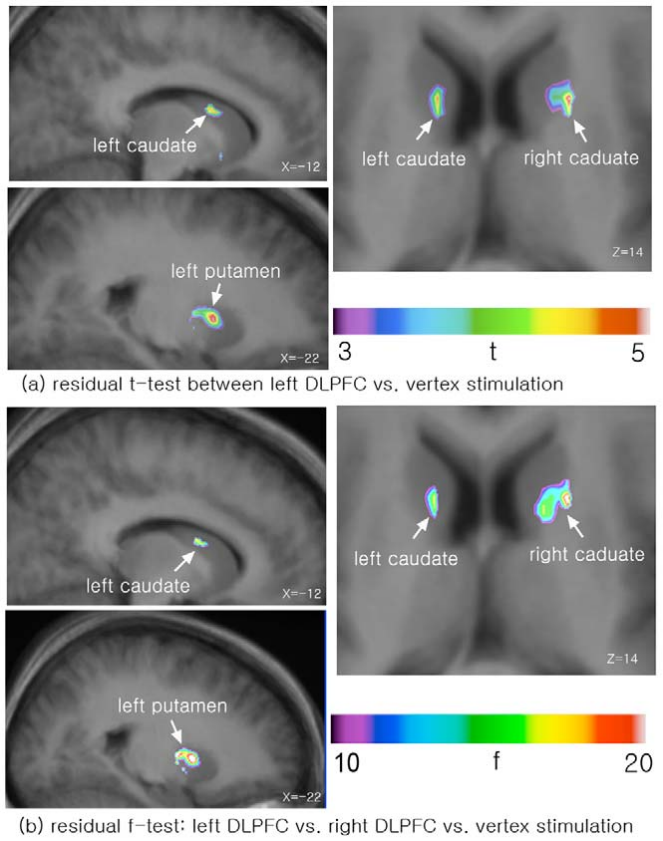
The similar results of t-test and f-test on real data. (a) t-test between left DLPFC stimulation vs. vertex stimulation (control condition): The t-map is overlaid upon the averaged MRI of all subjects in standard MNI space (published with permission from Ko et al. [9]). (b) f-test of all three stimulation condition (left DLPFC, right DLPFC and vertex stimulation): As the right DLPFC stimulation had no significant effect on BP in these regions, the f-map shows the similar pattern of t-map of left DLPFC vs. vertex stimulation.

Our previously published data [9], when re-visited with the residual f-test, revealed that the residual f-test would have confirmed our significant effect of cTBS on task-induced dopamine release in the left caudate (X = −12, Y = 8, Z = 14, f = 17.9, p<0.001 corrected, Table 3), right caudate (X = 20, Y = 8, Z = 14, f = 23.8, p<0.001 corrected) and left putamen (X = −22, Y = 6, Z = 2, f = 24.0, p<0.001 corrected), i.e., the same regions that were previously reported after using the *t-test* between left DLPFC and vertex stimulation (Fig. 2) [9]. In contrast, the conventional f-test did not detect any significant voxels at p<0.05 (corrected).

**Table 3:** Peak-cluster identified in the left caudate nucleus using different f-tests from real [^11^C]raclopride PET study [9].

	p-threshold	f-threshold	Max f	number of voxels
residual f-test	<0.001 corr.	15.3	17.9	4
conventional f-test	<0.05 corr.	20.8	7.5	0
	<0.001 uncorr.	10.4	7.5	0

**Figure 3:**
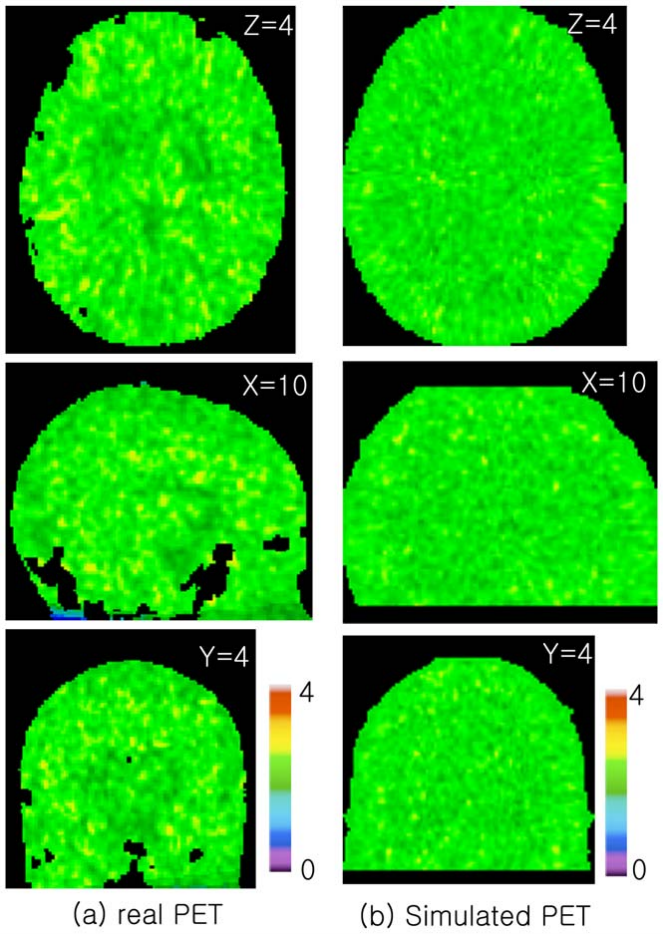
Averaged Durbin-Watson statistics-map over 10 subjects with (a) real [^11^C]raclopride PET and (b) simulated [^11^C]raclopride PET. All PET images from both real and simulated data passed the Durbin-Watson test, i.e., the statistics were >1.65 [21].

No autocorrelation of residuals were detected as the Durbin-Watson statistic was higher than its upper limit (>1.65, k = 3, n = 30) in all simulated and real data in the striatum (Fig. 3).

## Discussion

We have demonstrated the superior sensitivity of a residual f-test on simulated data as compared to the conventional f-test. Qualitatively, the f-statistics were not very different between the two methods (Table 1), but the greater degree of freedom of the residual f-test lowered the corrected threshold for significance. Indeed, using the conventional f-test, the number of voxels identified to have significant f-values was increased when more subjects were included in the analysis. However, this trend was not observed when using residual f-test, possibly due to the ceiling effect of the high degree of freedom utilized in the residual f-test, which in theory allows us to perform the test on a smaller number of subjects [2]. However, in practice, the number of subjects required for valid statistical tests should be determined after considering the test-retest variability, counter-balancing of scan orders and other underlying noise that may present. Nevertheless, neither method detected any false positives in the simulation study at the given p-threshold (p<0.001 corrected for residual f-test; p<0.05 corrected for conventional f-test; Table 2), thus we propose that the residual f-test can be applied successfully to increase the sensitivity of receptor-ligand PET studies without generating false positives.

It must be noted that when the threshold for significance in the residual f-test is lowered to p<0.01, some false positives were identified when only six subjects were included in the analysis, while the conventional f-test did not (Table 2). This may suggest that the specificity of the residual f-test is not as robust as the conventional f-test. However, the sensitivity of the conventional f-test is considerably lower than residual f-test, as it failed to detect significant f-statistics when the changes in BP were subtle (Table 1). Nevertheless, in order to compensate for the lower robustness of the residual f-test in controlling type 1 error compared to the conventional f-test, we recommend thresholding p at <0.001 rather than p<0.05 to determine significant voxels when using the residual f-test. This practice will ensure greater control of false positives while preserving the high sensitivity of the residual f-test.

The superior sensitivity of residual f-test when only a small change in BP (i.e., 5–10%) was induced is of particular interest to many PET imaging researchers. In fact, a recent review of PET studies that investigated behavior-induced dopamine release summarized that a third of the reviewed studies reported less than 10% change in the radioligand BP [24]. While increasing the sample size may provide enough statistical power to detect small changes in BP, it is desirable to also to try to minimize the sample size not only for research costs but also for ethical reasons.

Using the residual f-test we were able to successfully replicate our previous study [9] in which we showed only left DLPFC stimulation increased [^11^C]raclopride binding while right DLPFC stimulation did not compared to vertex stimulation. As the right DLPFC stimulation had no significant effect on BP in the striatum, the f-map (Fig. 2b) shows a similar pattern to the t-map, i.e. left DLPFC vs. vertex stimulation (Fig. 2a). In contrast, the conventional f-test could only detect significant voxels if no correction for multiple comparisons was applied [9].

The residual f-test extends the residual t-test, proven to be more sensitive than the conventional t-test [2], for cases in which more than two conditions are included in the study design. Therefore, not only does it have the same advantages that the residual t-test has, but also its limitations, e.g., motion related artifacts and inter-subject variability in anatomy and baseline BP. Although we tried to maximize the influences of realistic variables into the simulation, real cerebellar TACs and real anatomical segmented MRIs, it was not possible to employ motion artifact by using PET-SORTEO. Head motion between PET frames may introduce more fluctuation in the TAC, which may increase the residuals of model fitting [3]. This may reduce the f-values in the residual f-test, but not in the standard f-test. However, head movement between frames can be easily overcome by employing frame-by-frame motion correction methods in the analysis [25], [26]. On the other hand, head motion within PET frames may over- or under-estimate TACs depending on the shape of movement and regions, and it is difficult to predict its effect on either type of f-statistics.

We chose to simulate [^11^C]raclopride since it is the most widely used neuroreceptor ligand that has been used to investigate neurotransmitter displacement [1], [24]. In addition, we evaluated the feasibility of utilizing residuals in the t or f-statistics and confirmed no presence of autocorrelation for [^11^C]raclopride PET (Fig. 3). Although there are no obvious reasons not to extrapolate the usage of residual f-test toward other ligands, one should consider the possibility that differences in kinetics may affect the computing of the standard deviation of BP from the residuals of the model fitting [2]. Nonetheless, the superiority of the residual f-test may be even more valuable in certain neuroreceptor ligand PET studies whose search regions are beyond the small structures such as the striatum, e.g., [^11^C]FLB 457 [8], [27], [28], since these require a greater degree of correction for multiple comparisons [22], [29].

Given that we used PET–SORTEO, a realistic PET simulator based on real individual MRIs [19], [20], the role of partial volume effects (PVE) should also be taken into consideration [30]–[32]. The PVE may have broadened but lowered the effect of varying BPs in simulation. For example, when 20% changes in BP were simulated with 10 subjects, the cluster sizes that were identified by both statistical tests were >100 while only 99 voxels were manipulated (Table 1). Several methods have been proposed for correcting PVE [33]. Therefore, further studies with different PVE correction methods are encouraged to potentially increase the sensitivity of the residual f-test.

The input TAC was extracted from the cerebellum of the real PET scans [9], thus the TAC simulation of other brain regions (Eq. 3) are likely contaminated by partial volume effect. This may have reduced the signal-to-noise ratio and resulted in less realistic simulation of dynamic PET images. However, the cerebellum is fairly a large structure and we carefully delineated the cerebellum to only include grey matter. It has been previously reported that the recovery coefficient for 13.0 mm-diameter cylindrical phantom is higher than 90% when the image is reconstructed with 5 mm full-width-half-maximum ramp filter [30]. Therefore, the partial volume effect introduced by the use of real cerebellar TAC might have been insignificant.

The PET-SORTEO simulation was performed on each individual's segmented MRIs after transformation into the standard space to simplify the simulation procedures (c.f., [34], [35]). This may have reduced the realistic errors of co-registration/normalization of simulated PET images, which may have improved the signal-to-noise ratio compared to real PET experiment. However, the conventional f-test was also performed on the same PET images, therefore both types of f-tests were compared at the same level of noise environment.

In conclusion, we propose a “residual f-test”, based on the residual t-test [2], for use in PET study designs involving more than two conditions. This test allows us to take advantage of larger degrees of freedom than afforded when using the standard f-test, and therefore mitigates the increased susceptibility to type 2 errors inherent to PET studies with small sample sizes.
